# Correction: Biofunctional Janus particles promote phagocytosis of tumor cells by macrophages

**DOI:** 10.1039/d2sc90135h

**Published:** 2022-07-01

**Authors:** Ya-Ru Zhang, Jia-Qi Luo, Jia-Xian Li, Qiu-Yue Huang, Xiao-Xiao Shi, Yong-Cong Huang, Kam W. Leong, Wei-ling He, Jin-Zhi Du

**Affiliations:** Guangzhou First People's Hospital, Institutes for Life Sciences, School of Medicine, South China University of Technology Guangzhou 510006 China djzhi@scut.edu.cn; School of Biomedical Sciences and Engineering, South China University of Technology Guangzhou International Campus Guangzhou 510006 China; National Engineering Research Center for Tissue Restoration and Reconstruction, Key Laboratory of Biomedical Materials and Engineering of the Ministry of Education, Key Laboratory of Biomedical Engineering of Guangdong Province, Innovation Center for Tissue Restoration and Reconstruction, South China University of Technology Guangzhou 510006 China; Department of Gastrointestinal Surgery, The First Affiliated Hospital, Sun Yat-sen University Guangzhou Guangdong 510080 China hewling@mail.sysu.edu.cn; Guangzhou Regenerative Medicine and Health Guangdong Laboratory Guangzhou 510005 China; Department of Biomedical Engineering, Columbia University New York NY 10027 USA

## Abstract

Correction for ‘Biofunctional Janus particles promote phagocytosis of tumor cells by macrophages’ by Ya-Ru Zhang *et al.*, *Chem. Sci.*, 2020, **11**, 5323–5327, https://doi.org/10.1039/D0SC01146K.

The authors regret an error in Fig. 4a, where two of the panels contain partial overlap.

The panels for 2 h SPA3 and 2 h Tf + aSIRPα + SPA3 contain overlap, as the wrong data was initially used for 2 h SPA3. An independent expert has viewed the new data and has concluded that it is consistent with the discussions and conclusions presented.

The correct Fig. 4 is shown below.

**Fig. 1:**
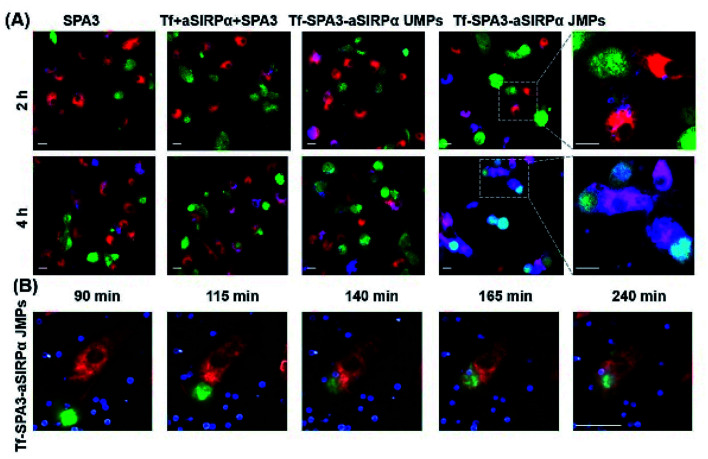
Tf–SPA3–aSIRPα JMPs promote the interaction and subsequent phagocytosis of B16F10 cells by BMDMs. (A) Representative confocal images of phagocytosis assays treated with different formulations for 2 or 4 h, respectively. (B) Time-dependent of phagocytosis treated with Tf–SPA3–aSirpα JMPs. In (A) and (B), B16F10 cells were labelled with CFSE (green), BMDMs were labelled with eFluor 670 (red) and particles were labelled with RB (blue). Scale bar: 20 μm.

The Royal Society of Chemistry apologises for these errors and any consequent inconvenience to authors and readers.

## Supplementary Material

